# Nuss Technique for Pectus Excavatum in Adult Patients: Cosmetic Satisfaction and Improvement of Quality of Life in a Single-Center Experience

**DOI:** 10.3389/fsurg.2022.903791

**Published:** 2022-06-01

**Authors:** Domenico Viggiano, Stefano Bongiolatti, Sara Borgianni, Roberto Lo Piccolo, Luca Voltolini, Alessandro Gonfiotti

**Affiliations:** ^1^ Thoracic Surgery Unit, Careggi University Hospital, Florence, Italy; ^2^ Department of Pediatric Surgery, University of Florence and Children’s University Hospital A. Meyer, Florence, Italy

**Keywords:** pectus excavatum, Nuss procedure, pectus bar, chest wall deformities, minimally invasive repair of pectus excavatum

## Abstract

**Objectives:**

Since its introduction, the Nuss minimally invasive procedure for pectus excavatum (PE) repair (MIRPE) has become the method of choice. The current study describes our experience of PE correction in adults, with particular focus on postoperative outcomes, pain, quality of life, and patients’ satisfaction.

**Methods:**

We enrolled for this observational study *n* = 93 adult patients from 2011 to 2018. The Haller index was used to quantify PE severity. Pulmonary function tests and cardiac examinations were performed preoperatively; we developed a standardized surgical technique and postoperative treatment, including follow-up at 3, 12, and 24 months after surgery and 6 months after bar removal. We also evaluated the quality of life and the satisfaction with the cosmetic result after the procedure with standardized questionnaires.

**Results:**

No operative or perioperative deaths occurred nor life-treating complications. Thirteen complications occurred in 12 patients, with a total complication rate of 14% (*n* = 13/93). Pain intensity decreased in the follow-up [pain score visual analog scale at 3 months: median 1 (0–8); 12 months: median 1 (0–5); and 24 months: median 1 (0–4)]. Better or much better quality of life after the Nuss procedure was observed: *n* = 79 (84.1%) at 3 months, *n* = 80 (86%) at 12 months, and *n* = 85 (91.4%) at 24 months. After 2 years of observation, more than 90% of patients described improvement in their quality of life and satisfaction with the cosmetic results. Only a very small group of patients suffered from pain in the follow-up.

**Conclusion:**

Our results demonstrate that the MIRPE procedure is safe and can be performed with excellent results in adults both for improvement of quality of life and for satisfaction with cosmetic results.

## Introduction

Pectus excavatum (PE), or “funnel chest,” constitutes 90% of all chest wall deformities and is reported in about 1:400 and 1:1,000 live births. It affects males in a rapport 4:1 to females and is typically present in early childhood, tending to become more pronounced during pubertal growth (1). The most common symptoms associated with the chest abnormality include dyspnea (especially with exercise), easy fatigability, and chest pain (2). Body image embarrassment, which results in negative psychological impact and low quality of life, is often the main worry for patients (3). However, reducing PE only as a cosmetic disorder is not completely proper: a short life expectancy was found by Kelly and colleagues reviewing autopsies of patients affected by PE (4) and also a reduced pulmonary function was associated with severity of abnormality (5, 6).

In 1998, Dr Donald Nuss introduced a minimally invasive repair of PE (MIRPE), temporarily implanting metal bars to modify the curvature of the anterior chest wall. This procedure easily gained consensus among surgeons and quickly established itself (7), revolutionizing the management of this chest deformity.

Children and adolescents are considered the ideal candidates for this approach according to the principle of this technique (8), while the rigidity/stiffness of the adult chest wall and its limited theoretical remodeling favored, for these patients, the use of a more invasive traditional Ravitch procedure.

In recent years, many authors published encouraging results about MIRPE in the adults, highlighting the feasibility and safety of the procedure (9, 10) and supporting the improvement of the cosmetic appearance after bar removal (11): the number of adults considered for PE repair increase and so more strong evidence is necessary to help surgeons in counseling about risks and benefits of MIRPE in this cluster.

Our work aims to report the results of a single-institution experience in the management of PE with the Nuss procedure in adults by evaluating mid-term outcomes and overall patient satisfaction after bar removal.

## Patients and Methods

From January 2011 to December 2018, we retrospectively reviewed the medical records of all patients aged 18 years or older who underwent MIRPE at the University Hospital of Florence. Our institutional review board granted approval and waived the requirement for specific informed consent for this retrospective study.

We excluded patients with a history of connective tissue disorders, recurrent PE, and asymmetric and combined deformity and also those who underwent combined cardiothoracic procedures.

Indications for surgery were both clinical and cosmetic reasons.

Preoperative evaluation included clinical examination, static or dynamic pulmonary function tests, cardiac evaluation with echocardiography, and chest computed tomography scan. The Haller index (HI) was used to quantify PE severity (12) and determined the main indication for PE surgical correction, but a low HI value was not considered a contraindication for MIRPE if a psychosocial disability is present.

### Surgical Technique

All operations were performed under general anesthesia using a double-lumen tube for selective single-lung ventilation. The patient was positioned supine on the operating table, with the chest elevated, using several blankets to allow the arms adducted in a lower position. The most depressed area of the sternal plate and hinge points on both sides of the chest ridge was identified and marked. A 5-mm thoracic port was bluntly introduced in a latero-posterior right position to identify the deepest point of PE, and carbon dioxide insufflated at 4–6 mm Hg pressure was used to find and dissect the safe retrosternal space.

Two curved lateral incisions, 3–4 cm long, were made just at the inferior edge of pectoralis major muscles, and a subcutaneous plane was created. An introducer was inserted into the right chest at the selected intercostal space, and the dissection was done under vision just above the pericardium. Once the left side of the desired intercostal space was reached, the tip of the introducer was pushed through the intercostal space. One side of a 40-cm-long polyvinylchloride suction connecting tube (Extrudan Surgery, Birkerod, Denmark) was plugged into the introducer tip, and the other side was connected to the right end of the curved bar (13). The introducer was carefully pulled backward from left to right, followed by the tube, creating a path for the bar to pass with the concave side up; then, the bar was rotated 180° around its long axis.

The entire procedure was completed smoothly and easily in a few seconds under thoracoscopic view. The use of one or two bars depended on the intraoperative cosmetic result; usually, we inserted the proximal bar first. Until 2011, we placed two stabilizers bilaterally to avoid rotation of the bar, but then, we placed only one stabilizer on the left side to reduce pain and wound troubles. After the procedure, we placed one chest tube on the right, usually removed on the first postoperative day. All patients were treated with non-steroidal anti-inflammatory drugs, paracetamol, and, if necessary, opioids (MS Contin controlled-release morphine or OxyContin oxycodone) by mouth for 3–6 weeks.

### Clinical Evaluation and Follow-Up

We organized in our outpatient department a routine evaluation for each patient at 3, 12, and 24 months after surgery and 6 months after bar removal. The bars were usually removed after 32–40 months.

Satisfaction with the cosmetic result, impact of surgical repair on the quality of life, and pain were assessed with two specific questionnaires: the first included two parts derived from the Nuss and Krasopoulos questionnaire in a modified format as presented in Table 1 (9, 14, 15), and the second was the DN4 questionnaire for neuropathic pain (16).

**Table 1:** Patients’ characteristics and perioperative course.

Sex M/F, %	85/8 (91.4/8.6)
Age at surgery (median, range)	23 (18–42)
Haller index (mean, range)	5.1 (2.3–12.6)
FEV1 (mean, range), L	3.8 (19–5.3)
FVC (mean, range), %	89.6 (58–115)
Operative time (median, range), min	45 (35–95)
Hospitalization (median, range), days	7 (5–13)
Number of bars inserted	One in 69 patients (74.2%) Two in 24 patients (25.8%)

*M, male; F, female; FEV1, forced expiratory volume 1; FEV, forced vital capacity.*

### Statistics

Continuous variables are expressed as mean or median value and range. Categorical variables are reported as counts and percentages. All data were analyzed with the use of SPSS software (version 24, SPSS Inc., Chicago, IL, USA).

## Results

### Patients’ Characteristics

Ninety-three patients underwent MIRPE between 2011 and 2018 with a median age at operation of 23 years (range 18–42). The majority were male (85 of 93 patients, 91.4%), and the mean HI was 5.1 (range 2.3–12.6). Chest pain, dyspnea, and decreased tolerance to physical exercise were the most frequent symptoms and were reported in 55 (59.1%) patients. Eighty-six patients (92.4%) referred to a poor self-image with a tendency to avoid social situations in which they have to show their deformity.

Patients’ characteristics and surgical results are reported in Table 1.

One bar was adequate for the correction of PE in most patients (*n* = 69/93; 74.2%).

### Complications and Bar Removal

**Figure 1:**
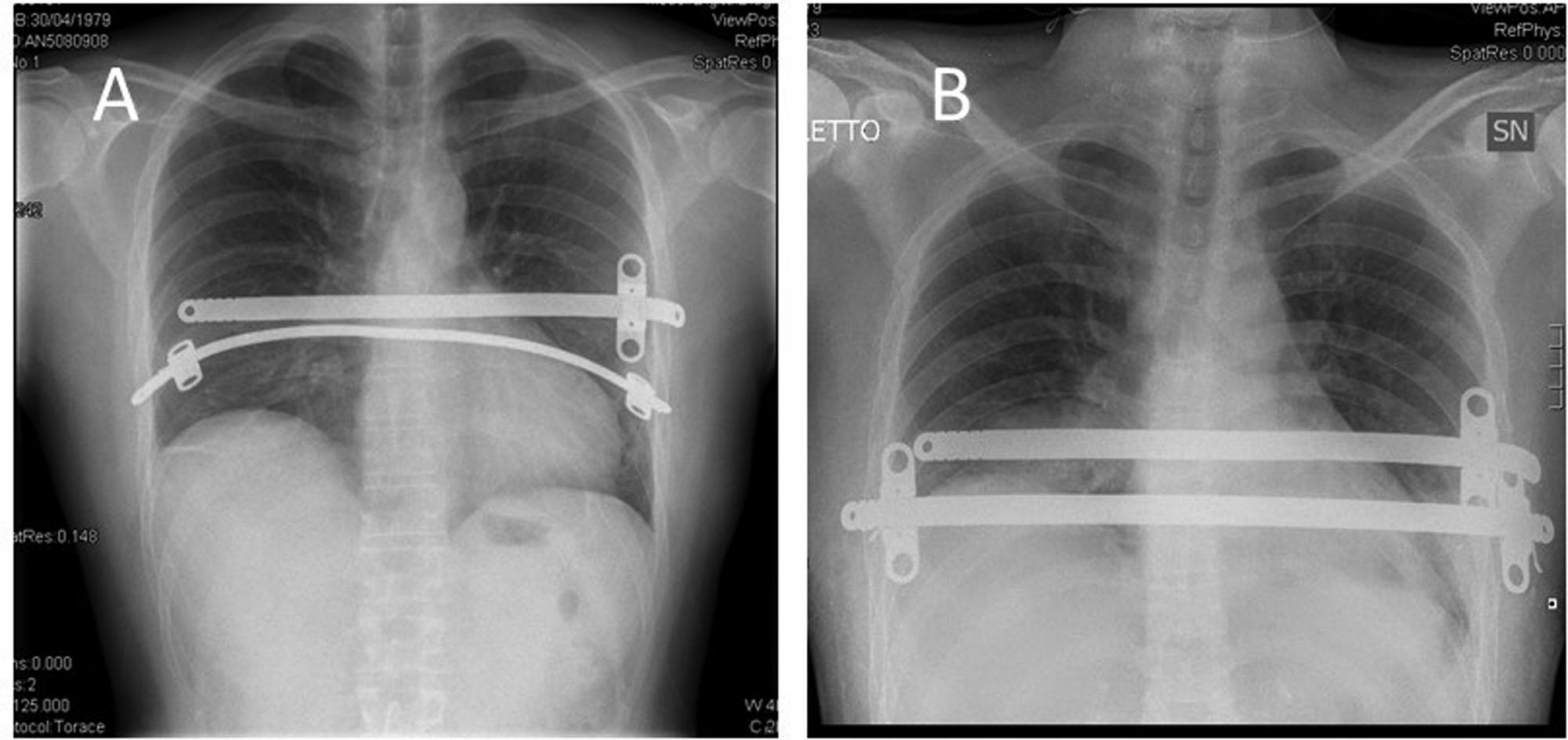
Patient with a dislocation of a bar (**A**) and with the bar in the correct position after surgical revision (**B**).

No operative or perioperative deaths occurred nor life-treating complications. Thirteen complications developed in 12 patients (12.9%): seroma/hematoma *n* = 2 (2.1%), wound infection *n* = 2 (2.1%), hemothorax *n* = 1 (1.1%), pneumothorax requiring chest tube reinsertion *n* = 4 (4.3%), and bar displacement *n* = 4 (4.3%) (Figure 1). Bar displacement always needed a surgical revision; all these re-operations were performed thoracoscopically.

Bar removal was carried out between 32 and 40 months from the first operation, with a median period of 34 months. All the bar removal procedures were uneventful. No PE relapse was observed after bar removal.

### Follow-Up and Mid-Term Results

We evaluated all 93 patients in the follow-up at 3, 12, and 24 months after surgery and 6 months after bar removal (Table 2). The median of follow-up was 39 months after surgery (range 38–46 months); the last patient was registered at follow-up in January 2022. High levels of satisfaction were registered: 97.8% of patients were satisfied or very/extremely satisfied with the cosmetic results 3 months after the operation. In the mid-term evaluation, this result was stable with 97.8% and 96.7% at 12 and 24 months, respectively, after surgery. At 6 months after bar removal, the percentage of satisfaction for cosmetic results was 97.8%.

**Table 2:** Results of the satisfaction questionnaire after minimally invasive repair of pectus excavatum (MIRPE).

Satisfaction (no. of patients), %	Extremely satisfied	Very satisfied	Satisfied	Moderately dissatisfied	Dissatisfied
3 months after surgery	(32) 34.4	(44) 47.3	(15) 16.1	(2) 2.2	(0) 0
12 months after surgery	(37) 39.8	(41) 44.1	(13) 13.9	(2) 2.2	(0) 0
24 months after surgery	(51) 54.8	(33) 35.4	(6) 6.5	(2) 2.2	(1) 1.1
6 months after bar removal	(54) 58.1	(33) 35.4	(4) 4.3	(1) 1.1	(1) 1.1

Better or much better quality of life after MIRPE was observed in 84.9% of patients at 3 months, 86% of patients at 12 months, 91.4% of patients at 24 months, and 94.7% of patients at 6 months after bar removal (Table 3). Three months after the operation, 53 patients (56.9%) reported no pain [visual analog scale (VAS) = 0], while in the remaining 40 patients, the pain intensity decreased during the follow-up. Pain scores are shown in Tables 4, 5. The pain was referred to as torsion movements and was the main cause of unchanged or worsened quality of life 1 year after the operation in 13 patients (13.9%). Only eight patients (8.6%) were disturbed by the bar after 24 months (pain in case of work-related movements); five of them did not need painkillers.

**Table 3:** Results of the quality-of-life questionnaire after MIRPE.

Quality of life (no. of patients), %	Much better	Better	Same	Worse
3 months after surgery	(44) 47.3	(35) 37.6	(9) 9.7	(5) 5.4
12 months after surgery	(52) 55.9	(28) 30.1	(8) 8.6	(5) 5.4
24 months after surgery	(63) 67.7	(22) 23.7	(4) 4.3	(4) 4.3
6 months after bar removal	(65) 69.9	(23) 24.8	(3) 3.2	(2) 2.1

**Table 4:** Pain intensity after MIRPE.

	VAS 0–10	
	Median	Range
3 months after surgery	1	0–8
12 months after surgery	1	0–5
24 months after surgery	1	0–4
6 months after bar removal	0	0–2

**Table 5:** Neuropathic pain after MIRPE.

	DN4 0–10	
	Median	Range
3 months after surgery	1	0–7
12 months after surgery	1	0–3
24 months after surgery	0	0–2
6 months after bar removal	0	0–2

At 24 months, more than 90% of patients described improvement in their quality of life. Nevertheless, cosmetic satisfaction was very high, with >90% of patients being satisfied: only four patients (4.3%) were not satisfied with the presence of hypertrophic skin scarring. These results remain stable also 6 months after bar removal with 94% of patients’ satisfaction: in addition, two of the four patients dissatisfied at 24 months after surgery became very satisfied after bar removal because of plastic reconstruction of previous hypertrophic scar.

## Discussion

The etiology of PE is uncertain, but a familial tendency was found in clinical experience. We also found some positive family history with similar diseases and other connective tissue diseases in our patient series. Other studies showed that the etiology remains unclear, but it appears to be polygenetic.

Abnormal skeletal growth with inward compression of the sternum upon intra-thoracic structures is the main pathophysiological mechanism in this illness.

Other researchers said, it is formed as a result of unbalanced growth of the costochondral regions of the anterior chest wall, leading to symmetric and asymmetric anomalies.

The diagnosis of PE is clinical, but a chest CT scan had a central role in the clinical assessment of PE and severity in measuring the HI.

In our study, the MIRPE procedure was suggested in cosmetically unsatisfied patients and/or with significant psychological impact, while the percentage of symptomatic PE was low. Patients often have body image embarrassment, which may result in adverse psychological symptoms and lower quality of life (2, 3). PE is often considered a purely cosmetic disorder; Kelly and colleagues reviewed autopsies and concluded that patients with PE have a shorter life expectancy because of that surgical repair in an adult is worthy and requires more attention (4).

The correction of PE in adults is a challenging procedure (9) because the rigid chest wall is less pliable than in childhood.

For the differences in costal cartilage flexibility, there is a biomechanical disadvantage in performing the Nuss procedure in adults (17, 18). Previous studies reported a linear relationship between age-related rigidity and the elevating force needed to raise the depressed sternum, approximately 2–3 times greater than that in children (19). These physiological reserves, however, have not been frequently addressed in the literature: in fact, long-term results are rare, and there is a lack of prospective randomized trials comparing MIRPE with conventional surgery (e.g., Ravitch approach) (20).

Kim and colleagues (21) demonstrated longer operation time and higher complication rate for MIRPE in adults compared with children (58.3% vs. 11.1%); the same authors recommended a careful selection of the patients to accomplish better outcomes.

In the present series, all patients were adults with a median age of 23 years (range 18–42): mortality was zero and we did not observe any severe injury during surgical correction; we had 12 patients who developed 13 postoperative complications (12.9%), a rate similar to previous reports (22, 23). We experienced four-bar dislocations, all due to laceration of the intercostal space since the extreme chest wall rigidity. To overcome this inconvenience, we applied a longer bar, positioned over the laceration in such a way that the stabilizer was inserted in an intact intercostal space.

In adults, because of the presence of a relevant stiffness of the anterior chest wall, it is often necessary to insert two bars to correct the defects. In fact, Pilegaard et al. and Hoksch et al., in their recent works (9, 23), reported an incidence rate of 19%–37% of placement of two bars for optimal correction. Similar data were presented in our series: 24 out of 93 patients (25.8%) needed two bars for correction without any added complication and with good cosmetic results, as demonstrated by the questionnaire applied.

Nagasao et al. (24) described that the insertion of two bars might cause less pain due to a better distribution of the dynamic forces at multiple thoracic levels; our outcomes confirmed this observation: in fact, at 24 months of follow-up, our patients experienced a median pain VAS score of 1 (range 0–4). Another inconvenience of the two bars was the increased operative time to removal.

MIRPE has shown a positive effect on the psychosocial well-being of children with PE and also an improvement in all indicators of psychosocial functioning, including body image satisfaction, as described by Lawson and colleagues (6). A similar positive effect was shown in young males by Krasopoulos et al. (14). Our series confirmed these results of significant improvement in the quality of life in the immediate postoperative period but also distant from surgery at 24 months and 6 months after bar removal.

The limitations of this study include all the potential biases proper of all retrospective analyses. In particular, it is a single-institution series analyzed between 2011 and 2018. Furthermore, data concerning the cosmetic appearance and postoperative pain are subjective, with high variability between patients and then not universally valid and standardized.

## Conclusion

This paper demonstrated that MIRPE is a feasible approach for PE correction in adults and is associated with good cosmetical and subjective improvement of quality of life. The Nuss technique ensures excellent results in terms of chest wall remodeling, showing a high level of patient satisfaction in the early and mid-term period and also after bar removal.

In the future, more rigorous long-term studies are needed to better explain the role of MIRPE in adults.

## Data Availability

The raw data supporting the conclusions of this article will be made available by the authors without undue reservation.

## References

[1] Feng J Hu T Liu W Zhang S Tang Y Chen R The biomechanical, morphologic, and histochemical properties of the costal cartilages in children with pectus excavatum. J Pediatr Surg. (2001) 36:1770–6. 10.1053/jpsu.2001.28820 11733904

[2] Kelly RE Jr. Pectus excavatum: historical background, clinical picture, preoperative evaluation and criteria for operation. Semin Pediatr Surg. (2008) 17:181–93. 10.1053/j.sempedsurg.2008.03.002 18582824

[3] Krille S Müller A Steinmann C Reingruber B Weber P Martin A. Self- and social perception of physical appearance in chest wall deformity. Body Image. (2012) 9:246–52. 10.1016/j.bodyim.2012.01.005 22366427

[4] Kelly RE Jr Lawson ML Paidas CN Hruban RH. Pectus excavatum in a 112-year autopsy series: anatomic findings and the effect on survival. J Pediatr Surg. (2005) 40:1275–8. 10.1016/j.jpedsurg.2005.05.010 16080931

[5] Swanson JW Avansino JR Phillips GS Yung D Whitlock KB Redding GJ Correlating Haller Index and cardiopulmonary disease in pectus excavatum. Am J Surg. (2012) 203:660–4. 10.1016/j.amjsurg.2011.12.013 22417849

[6] Lawson ML Mellins RB Paulson JF Shamberger RC Oldham K Azizkhan RG Increasing severity of pectus excavatum is associated with reduced pulmonary function. J Pediatr. (2011) 159:256–61.e2. 10.1016/j.jpeds.2011.01.065 21429515

[7] Antonoff MB Erickson AE Hess DJ Acton RD Saltzman DA. When patients choose: comparison of Nuss, Ravitch, and Leonard procedures for primary repair of pectus excavatum. J Pediatr Surg. (2009) 44:1113–8. 10.1016/j.jpedsurg.2009.02.017 19524726

[8] Hebra A. Minimally invasive repair of pectus excavatum. Semin Thorac Cardiovasc Surg. (2009) 21:76–84. 10.1053/j.semtcvs.2009.04.005 19632566

[9] Hoksch B Kocher G Vollmar P Praz F Schmid RA. Nuss procedure for pectus excavatum in adults: long-term results in a prospective observational study. Eur J Cardiothorac Surg. (2016) 50:934. 10.1093/ejcts/ezw130 27126132

[10] Pilegaard HK Licht PB. Routine use of minimally invasive surgery for pectus excavatum in adults. Ann Thorac Surg. (2008) 86:952–6. 10.1016/j.athoracsur.2008.04.078 18721589

[11] Pilegaard HK. Extending the use of Nuss procedure in patients older than 30 years. Eur J Cardiothorac Surg. (2011) 40:334–7. 10.1016/j.ejcts.2010.11.040 21232968

[12] Haller JA Kramer SS Lietman A. Use of CT scans in selection of patients for pectus excavatum surgery: a preliminary report. J Pediatr Surg. (1987) 22:904–6. 10.1016/S0022-3468(87)80585-7 3681619

[13] Messineo A Ghionzoli M Lo Piccolo R Milanez De Campos JR. A simplified method to pass the bar through the mediastinum in the Nuss technique. Ann Thorac Surg. (2015) 99(2):717–8. 10.1016/j.athoracsur.2014.09.072 25639424

[14] Krasopoulos G Goldstraw P. Minimally invasive repair of pectus excavatum deformity. Eur J Cardiothorac Surg. (2011) 39:149–58. 10.1016/j.ejcts.2010.07.019 20739187

[15] Krasopoulos G Dusmet M Ladas G Goldstraw P. Nuss procedure improves the quality of life in young male adults with pectus excavatum deformity. Eur J Cardiothorac Surg. (2006) 29:1–5. 10.1016/j.ejcts.2005.09.018 16337131

[16] Bouhassira D Attal N Alchaar H Boureau F Brochet B Bruxelle J Comparison of pain syndromes associated with nervous or somatic lesions and development of a new neuropathic pain diagnostic questionnaire (DN4). Pain. (2005) 114:29–36. 10.1016/j.pain.2004.12.010 15733628

[17] Fonkalsrud EW Reemtsen B. Force required to elevate the sternum of pectus excavatum patients. J Am Coll Surg. (2002) 195:575–7. 10.1016/S1072-7515(02)01245-0 12375767

[18] Nagasao T Miyamoto J Tamaki T Ichihara K Jiang H Taguchi T Stress distribution on the thorax after the Nuss procedure for pectus excavatum results in different patterns between adult and child patients. J Thorac Cardiovasc Surg. (2007) 134:1502–7. 10.1016/j.jtcvs.2007.08.013 18023673

[19] Nagasao T Miyamoto J Ichihara K Jiang H Jin H Tamaki T. Age-related change of postoperative pain location after Nuss procedure for pectus excavatum. Eur J Cardiothorac Surg. (2010) 38:203–8. 10.1016/j.ejcts.2009.12.047 20176494

[20] de Oliveira Carvalho PE da Silva MV Rodrigues OR Cataneo AJ. Surgical interventions for treating pectus excavatum. Cochrane Database Syst Rev. (2014) 2014(10):CD008889. 10.1002/14651858.CD008889.pub2 25352359 PMC6885037

[21] Kim DH Hwang JJ Lee MK Lee DY Paik HC. Analysis of the Nuss procedure for pectus excavatum in different age groups. Ann Thorac Surg. (2005) 80:1073–7. 10.1016/j.athoracsur.2005.03.070 16122489

[22] Hanna WC Ko MA Blitz M Shargall Y Compeau CG. Thoracoscopic Nuss procedure for young adults with pectus excavatum: excellent midterm results and patient satisfaction. Ann Thorac Surg. (2013) 96:1033–8. 10.1016/j.athoracsur.2013.04.093 23810179

[23] Pilegaard HK Licht PB. Early results following the Nuss operation for pectus excavatum—a single-institution experience of 383 patients. Interact CardioVasc Thorac Surg. (2008) 7:54–7. 10.1510/icvts.2007.160937 17951271

[24] Nagaso T Miyamoto J Kokaji K Yozu R Jiang H Jin H Double-bar application decreases postoperative pain after the Nuss procedure. J Thorac Cardiovasc Surg. (2010) 140:39–44. 10.1016/j.jtcvs.2009.12.027 20363484

